# ITGB2 fosters the cancerous characteristics of ovarian cancer cells through its role in mitochondrial glycolysis transformation

**DOI:** 10.18632/aging.205529

**Published:** 2024-02-11

**Authors:** Guo-Wei Li, Yan-Ping Jin, Jian-Ping Qiu, Xiu-Fang Lu

**Affiliations:** 1 Department of Rehabilitation Science, Nanjing Normal University of Special Education, Nanjing, Jiangsu 210000, China; 2 Department of Obstetrics and Gynecology, Zhongda Hospital Jiangbei Branch, School of Medicine, Southeast University, Nanjing, Jiangsu 210000, China; 3 Department of Obstetrics and Gynecology, Suzhou Municipal Hospital North, Suzhou, Jiangsu 215000, China

**Keywords:** glycolysis, mitophagy, malignant phenotypes, ovarian cancer

## Abstract

Related studies have shown that ITGB2 mediates mitochondrial glycolytic transformation in cancer-associated fibroblasts and participates in tumor occurrence, metastasis and invasion of cancer cells. Based on these studies, we tried to construct a mitochondrial glycolysis regulatory network and explored its effect on mitochondrial homeostasis and ovarian cancer cells’ cancerous characteristics. Our research revealed a distinct increase in the expression of ITGB2 and associated signaling pathway elements (PI3K-AKT-mTOR) in cases of ovarian cancer. ITGB2 might control mTOR expression via the PI3K-AKT pathway, thus promote mitochondrial glycolysis transformation and cell energy supply in ovarian cancer. This pathway could also inhibit mitophagy, maintain mitochondrial stability, and enhance the cancerous characteristics in case of ovarian cancer cells by mediating mitochondrial glycolytic transformation. Thus, we concluded that ITGB2-associated signaling route (PI3K-AKT-mTOR) may contribute to the progression of cancerous traits in ovarian cancer via mediating mitochondrial glycolytic transformation.

## INTRODUCTION

In 2020, there are almost 22,000 new ovarian cancer patients in USA, the number of deaths has nearly reached 14000. The 5-year survival percentage for ovarian cancer patients in China stands at 39.1%, while nearly 70% of patients will suffer recurrence within 1–2 years or even experience multiple recurrence. At the same time, the recurrence cycle may be shortened after each treatment with drug resistance [1, 2].

Ovarian cancer is described as a "silent killer" because of its high invasiveness and absence of distinct symptoms during its initial phase. The primary therapeutic approach for ovarian cancer cases involves a combination of surgery and chemotherapy. In recent years, due to continuous advances in treatment combined with individualized chemotherapy, the 5-year survival rate has significantly improved. However, with the emergence of platinum- and paclitaxel-resistant cases, the improvements in survival rate have slowed. Chemotherapy resistance and tumor recurrence are very common in late-stage cases. Currently, numerous academics hold the view that the drug resistance mechanism of ovarian cancer cells is involved in drug transport and metabolism, DNA damage repair, cell apoptosis, tumor autophagy and metabolic recombination [3].

Aerobic glycolysis (the Warburg effect) is an important metabolic pathway that maintains the malignant phenotype of tumor cells. It not only provides energy for its proliferation but also provides large numbers of intermediate products for the biosynthesis of tumor cells, thus assisting tumor cell immune evasion and enhancing the invasion ability of tumor cells. Therefore, the key regulatory molecules in the Warburg effect could provide new strategies and targets for malignant tumor therapy [4, 5]. Related studies have shown that ovarian cancer metabolism presents obvious heterogeneity, which is closely related to survival. However, in ovarian cancer, aerobic glycolysis and oxidative phosphorylation are activated, and several scholars have speculated that the progression of this disease might be intimately linked to this process.

β2 integrin is a leukocyte-specific heterodimer surface receptor that can participate in tumor cell adhesion matrix remodeling and signal transduction involving tumor cells and its surrounding microenvironment to trigger angiogenesis and distinct immune reactions. ITGB2 (CD18) is one of the key subunits of β2 integrin, and bioinformatic analysis revealed that ITGB2 expression was upregulated in high-risk groups and significantly correlated with the overall survival time in ovarian cancer patients. Several researchers believe that ITGB2 might be involved in the emergence and progression of ovarian cancer [6, 7]. Moreover, the PI3K-AKT-mTOR pathway is activated in various malignant tumors, and this route might play a role in the control of tumor cell proliferation, metastasis, survival, metabolism, autophagy and chemotherapy resistance. Related studies have shown that ITGB2 mediates mitochondrial glycolytic transformation in cancer-associated fibroblasts through the PI3K-AKT-mTOR pathway and participates in the occurrence, metastasis and invasion of oral squamous cancer cells [8–10]. Many academics hold the view that ITGB2 could encourage the advancement of ovarian cancer via PI3K-AKT-mTOR signalling pathway mediated mitochondrial glycolysis.

## MATERIALS AND METHODS

### Cell culture

The Institute of Chinese Academy of Sciences in Shanghai offered cell samples for this research (Ovarian Cells, SKOV3 and A2780). Every cell was grown in RPMI-1640 medium enriched with a mixture of 1% penicillin/streptomycin and 10% fetal bovine serum. The medium was replaced every three days while the cell samples were cultured at 37°C and 5% CO_2_ [11, 12].

### Clinical sample

For our investigation, a total of 120 clinical samples were used, comprising 39 ovarian samples and 81 samples of ovarian cancer. At the outset of this investigation, every case that provided clinical samples for this experiment signed the Informed Consent Statement. With 14 instances in stage I (FIGO Stage), 27 cases in stage II, 21 cases in stage III, and 19 cases in stage IV, the average age of patients in the ovarian cancer group was 41.33 ± 9.95 years. Furthermore, 39 patients who underwent a myomectomy at our hospital during the same time period and had normal ovarian tissue were chosen as the control group. In the NC group, the mean age of our cases was 43.21 ± 9.54 years. The age distributions of these two groups did not differ significantly (*P* > 0.05).

### Immunohistochemistry assay

In order to prepare paraffin sections and do immunohistochemistry staining, we obtained tissues from ovarian cancer and other ovarian diseases and fixed them in 10% neutral formalin. To make these tissues more visible, we first underwent dewaxing and dehydration. Subsequently, the dehydrated slides were purified using distilled water. Following antigen retrieval, the samples were sealed with serum, submerged in 3% H_2_O_2_ solutions for ten minutes, and subsequently the primary antibody was incubated at a 1:200 dilution throughout the night at 4°C. Following that, we gave these slides three PBS washes (for five minutes each), added horseradish peroxidase, and incubated them for thirty minutes at 37°C before giving them another PBS wash. After adding the reaction dye, the samples were cleaned, dried, and sealed with neutral glue. A microscope was used to examine the samples.

### Western blot analysis

We used RIPA protein lysate and PMSF to lyse a suitable number of clinical samples and ovarian cancer cells on ice. We next moved on to the electrophoretic transfer process. After adding the first antibody (1:1000), the specimens were allowed to incubate for an extra night at a temperature of 4°C. After adding the secondary antibody (1:5000), which was labeled with ECL and horseradish peroxidase, it was left to hatch in the dark room temperature for one hour. Lastly, we established GAPDH as the internal reference gene and examined the protein bands’ gray values using ImageJ (version 1.8.0).

### RIP assay

RIP tests were carried out in accordance with the directions using an immunoprecipitation kit. After choosing SKOV3 cells during their logarithmic growth stage, they were put to RIPA lysis solution, rinsed with PBS, and lysed for five minutes on ice. After removing the cell supernatant, the magnetic beads, layered with AGO2 and IgG, underwent incubation at 4°C for a whole night. After particular proteins were identified, we were able to get the protein-RNA complexes. Next, proteinase K was used to digest all of the samples in order to extract the RNA molecules. Ultimately, target gene expression was ascertained by isolating RNA samples and using PCR.

### FISH experiment (fluorescence *in situ* hybridization)

After being preheated to 37°C, 6 ml of KCl solution (0.075 mol/L) containing SKOV3 cells in optimal condition was added. The mixture was then placed in a water bath set at a continuous 37°C. We combined the sample with 2 milliliters of stationary liquid, allowed it to sit for 10 minutes at 37°C, and then removed it. Following centrifugation, we added 6 ml of fixed liquid and threw away the supernatant; we repeated this procedure several times before preparing and pipetting the cell suspension droplets. We conducted FISH studies on these materials after heating them to 75°C for 30 minutes. Following hybridization, all cell samples were put to a 1% NP-40 solution and rinsed for five minutes at 56°C using two times SSC buffer. Following their removal, the glass slides were dried, restained with 10 μl DAPI, and then examined under a fluorescence microscope. According to the test results, PI3K showed a red signal, ITGB2 showed a green signal, and the nucleus showed a blue signal.

### Cell culture and transfection

For the ITGB2 overexpression experimental group, we employed SKOV3 cells, and for the ITGB2 knockdown experimental group, A2780 cells. Through sgRNA cloning into the lentiCRISPR v2 vector and mixing with psPAX2 and PMD2.G, we were able to construct ovarian cancer cell types with distinct expression levels of ITGB2, PI3K, AKT, and mTOR RNA and protein. Subsequently, we sorted and planted 4 × 10^5^ ovarian cancer cells per well into 6-well plates in appropriate condition. We put the cells into 1.5 ml serum-free media with solution containing Lipofectamine 3000 once the cell density reached 70–80%. After 4–6 hours, the culture solution was swapped out for full media, and the plasmid was cotransfected into cell samples to generate lentivirus particles. Finally, we spent 48 hours transfecting them into SKOV3 and A2780 cells.

### Seahorse detection

Inoculating 2 × 10^4^ cells in Seahore Fe96 microporous plates, the cells were measured using a Seahore XF apparatus following treatment. Following the detection of basal respiration in a regulated state, an oligomycin ATP synthase inhibitor was applied. Following then, there was a noticeable decline in the oxygen consumption rate (OCR). Proton leakage was responsible for the remaining portion of the OCR reduction, which was brought on by oxidative phosphorylation. Following the addition of the uncoupling agent FCCP, the proton gradient will no longer be a constraint on the electron transfer’s maximal rate of operation. OCR will thus increase dramatically in order to achieve maximum oxygen consumption. Respiratory potential refers to the distinction it makes from basal respiration. In order to totally prevent electron transport and minimize the rate of oxygen consumption, we lastly added an electron transport inhibitor (such as antimycin A). Using the appropriate kits, lactic acid, proton leakage, and ATP contents were measured. The experimental procedures were followed precisely as directed by the manufacturer.

### Co-localization analysis

We took SKOV3 cells and seeded them at a density of 1 × 10^6^/well into 6-well plates, keeping them at 37°C and 5% CO_2_. Following that, we added 0.5% Triton X-100 for a 20-minute reaction and PFA for a 30-minute fixation. After adding goat serum and sealing for 30 minutes, we removed the sealing solutions, added the necessary quantity of primary antibody, and stored the containers at 4°C for the entire night. Following that, we added the appropriate fluorescent probes and secondary antibodies that were fluorescently labeled, and we incubated them for one hour at room temperature and in a dark environment. After adding DAPI and an anti-fluorescence quenching agent, they were examined under a fluorescent microscope.

### Ultrastructure observation using transmission electron microscopy (TEM)

After being injected into 6-well plates, SKOV3 cells were given a 24-hour treatment. After fixing these samples with 1% osmium tetroxide for two hours, cells underwent trypsin digestion and were stabilized using a 0.1 M phosphate buffer solution, enriched with 2% PFA and 2% glutaraldehyde. Following a one-hour (twice) simultaneous incubation with LR white resin, the cells underwent dehydration in a gradient composed of different ethanol concentrations (30, 50, 70, 90, and 100%), and then implanted into this resin. Uranyl acetate and lead citrate were used to dye the 60 nm-diameter sections of the cured blocks. A transmission electron microscope was used to examine each cell sample, and ultrastructure pictures were taken. Soochow University’s AMT imaging equipment was utilized to obtain digital images.

### Mitochondrial membrane potential (MMP) assay

In 6-well plates, logarithmic growth phase cells were plated at a density of 3 × 10^5^ cells/well and subjected to 24 hours of treatment based on the specific experimental groups. Next, using JC-1 probes in accordance with the guidelines to detect MMP in cell samples. Following JC-1 labeling, flow cytometry was used to evaluate the cell samples (529 and 590 nm), respectively, whereas the JC-1 excitation wavelength ranged at 488 nm.

### Proliferation assay

Cells that underwent transfection were distributed into plates with six wells each, and grown in media containing 10% FBS at 37°C and 5% CO_2_ for the colony formation test. Following a 10-day incubation period, each cell underwent staining using 0.1% crystal violet (Beyotime™, Haimen, China), and colony counts were done by hand. The transfected cells were cultivated in 96-well plates for the EdU experiment, and they were exposed to 100 μl of media containing 20 μM EdU. All cells were fixed with 4% paraformaldehyde (PFA) for 30 minutes, then treated with 0.5% Triton X-100 in PBS for 20 minutes. The nuclei were then stained with Hoechst for 15 minutes after the cells had been incubated at 37°C and 5% CO_2_ for two hours. Subsequently, the instructions were followed to compute the proliferation rate, and ImageJ was used to count and examine the cells in each vision area.

### Cell invasion assay

All samples used in the Transwell experiment were digested using 0.25% proteinase and centrifuged for five minutes at 1200 r/min. We next put Transwell chambers onto 24-well plates and added 5 × 10^5^/ml of cells to each well. Following that, we heated these plates for 20 to 30 minutes, added 800 milliliters of 4% PFA, twice-washed them in PBS, and then dyed them for 30 minutes with 0.1% crystal violet. Finally, the cells were observed and photographed using an inverted microscope to calculate and examine the number of cells passing through the ventricular membrane [13, 14].

### Xenograft animal study

Ten BALB/c nude mice (aged 21 days) were split into two groups at random: the NC group and the si-ITGB2 group. We subcutaneously injected 0.2 ml of SKOV3 cells (2 × 10^7^/ml) into the rear of each mouse in the NC group and 0.2 ml of ITGB2 knock-out SKOV3 cells (2 × 10^7^/ml) into the si-ITGB2 group. Each mouse received a single injection during this procedure, with the puncture point being fixed at 1.0 cm from the injection site. On the fortieth day, all mice harboring tumors were put to sleep through the administration of pentobarbital sodium via intra- peritoneal injection (150–200 mg/kg). The transplant tumors under the skin were then extracted, measured, stained, and examined.

### Statistical methods

Normal distribution measurement data were expressed as average ± variation (X ± S). The means of various groups were compared using variance analysis, and the means of two groups were compared using a *t* test. A result was deemed statistically significant if P was less than 0.05 (^*^p < 0.05, ^**^p < 0.01).

## RESULTS

### ITGB2 and its downstream signal pathway are upregulated in human ovarian cancer samples

**Figure 1:**
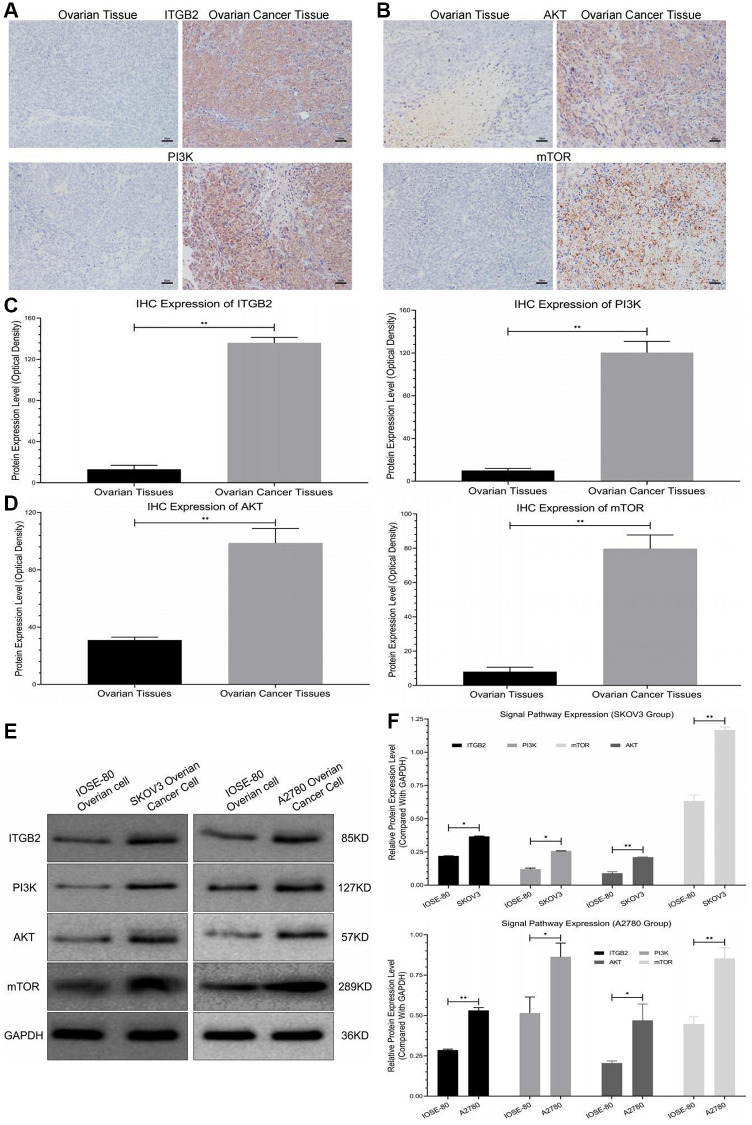
**Detection of signal pathway expression in clinical samples.** (**A**, **B**) Immunohistochemical detection of ITGB2-PI3K-AKT-mTOR in clinical samples (ovarian cancer and normal ovarian tissues). (**C**, **D**) Data statistics of signal axis Immunohistochemical detection in clinical samples. (**E**, **F**) WB detection of ITGB2-PI3K-AKT-mTOR in ovarian cancer cells (SKOV3 and A2780) and normal ovarian cells (IOSE-80).

Immunohistochemical staining revealed that the protein levels of the ITGB2 signaling pathway (PI3K-AKT-mTOR) were significantly higher in ovarian cancer tissues than in normal ovarian tissues (Figure 1A–1D). Moreover, we also evaluated this signal pathway regulatory factor expression in ovarian cancer cells (SKOV3-A2780) and normal ovarian cells (IOSE-80) using WB. The results revealed that ITGB2 and its downstream pathway factors (PI3K-AKT-mTOR) were obviously upregulated in ovarian cancer cells compared with normal ones (Figure 1E, 1F). Therefore, we concluded that the expression of ITGB2 and its related signaling pathway (PI3K-AKT-mTOR) were upregulated in ovarian cancer samples.

### Elevated ITGB2 in ovarian cancer patients positively correlated with poor prognosis

**Figure 2:**
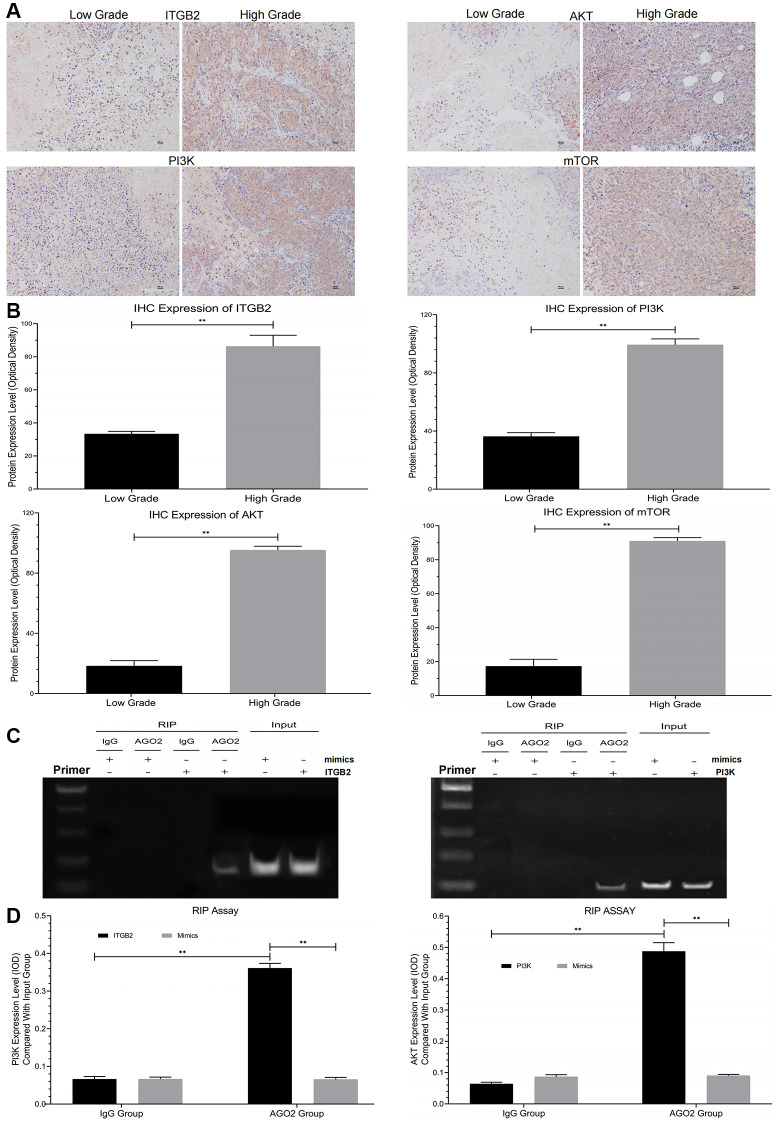
**Detection of signal pathway expression in clinical ovarian cancer samples and RIP assay.** (**A**, **B**) Immunohistochemical detection of ITGB2-PI3K-AKT-mTOR in different grades of ovarian cancer tissues (FIGO I-IV) and data statistics. (**C**, **D**) RIP assays of signal pathway factors (ITGB2, AKT vs PI3K) and data statistics.

Here, we used immunohistochemical staining to study the expression of ITGB2 and its related signal pathway (PI3K-AKT-mTOR) in different stages (FIGO I-IV) of ovarian cancer samples. The results showed that ITGB2-related signal pathway expression was significantly upregulated in high-grade ovarian cancer samples (FIGO III/IV) compared with low-grade ones (Figure 2A, 2B).

In this research, 41 cases were segregated into the Low Grade Group, while 40 cases were segregated into the High Grade Group. After standardized individual therapy, 2 cases suffered death in Low Grade Group after 5-year clinical follow-up, while 26 cases suffered death in High Grade Group. So, the 5-year survival rate was 95% in Low Grade Group and 60% in High Grade Group. Therefore, we concluded that high expression of ITGB2-related signal pathway in ovarian cancer patients is associated with their poor prognosis.

### ITGB2 plays regulatory roles through the PI3K-AKT-mTOR axis in ovarian cancer cells

**Figure 3:**
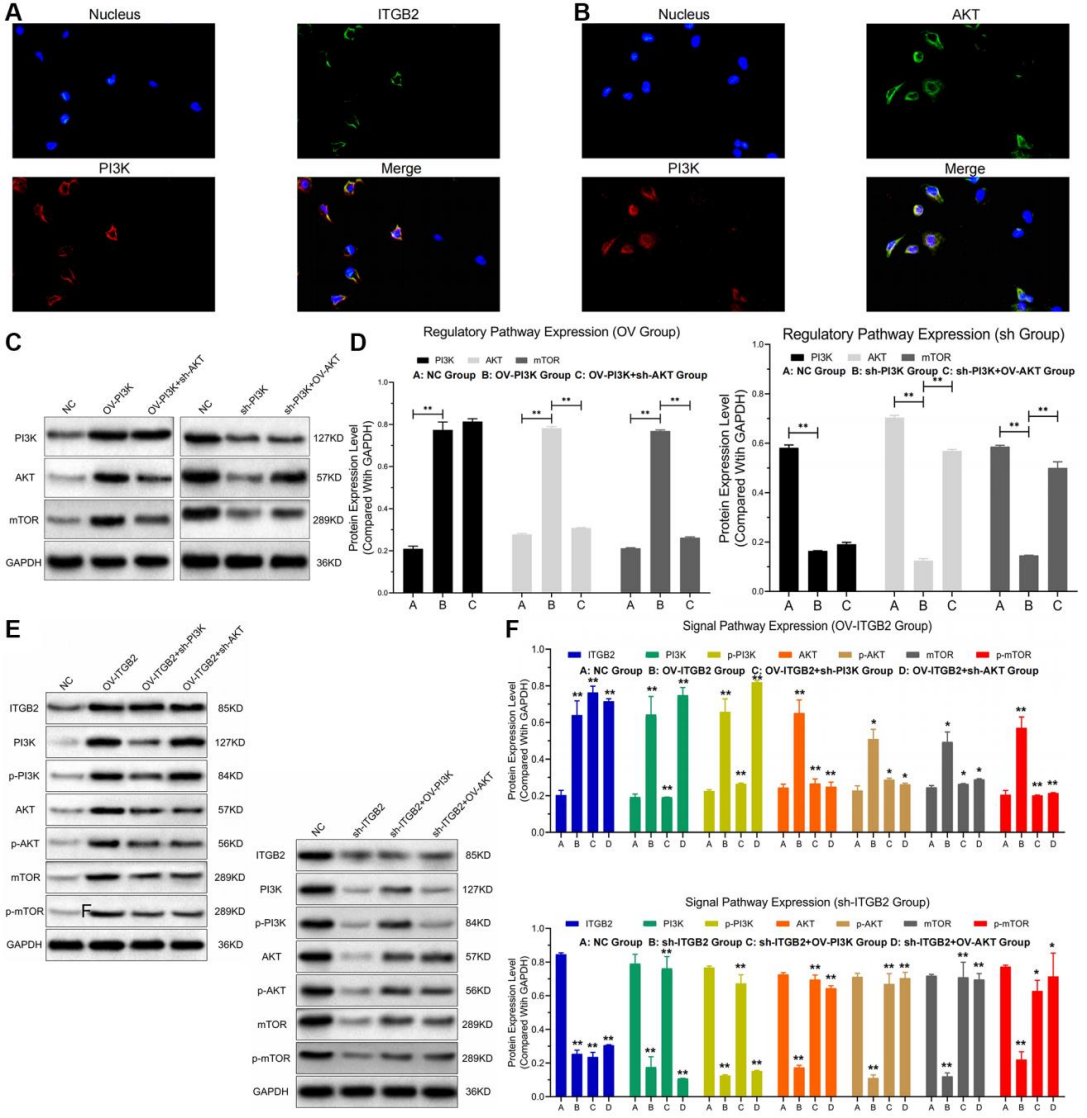
**ITGB2 related signal pathway regulation.** (**A**, **B**) FISH detection of signal pathway factors (ITGB2, AKT vs PI3K). (**C**, **D**) WB detection of ITGB2 related signal pathway expression in ovarian cancer cell models and data statistics. (**E**, **F**) WB detection of pathway factor phosphorylation expression and data statistics.

Analysis via RIP assays showed a notable rise in PI3K levels in ITGB2 samples exposed to AGO2, in contrast to those in the IgG group; additionally, AGO2 samples treated with ITGB2 exhibited notably elevated PI3K levels compared to those treated with mimics (Figure 2C, 2D). In the FISH experiment, we noticed that ITGB2-AKT (green) and PI3K (red) were situated within the cytoplasm of ovarian cancer cells (Figure 3A, 3B). Thus, we concluded that PI3K is a target for ITGB2 and AKT binding.

Then, we transfected ovarian cancer cells (SKOV3 and A2780) with lentivirus plasmids and detected the expression of PI3K, Akt and mTOR at the protein and phosphorylation levels through WB assays. Findings indicated that the protein concentrations of PI3K (pi-PI3K), AKT (pi- AKT) and mTOR (pi-mTOR) were significantly increased in ITGB2-overexpressing ovarian cancer cells (SKOV3), and these increases could be reversed after PI3K and AKT inhibition. In ITGB2- silenced ovarian cancer cells (A2780), the protein levels of PI3K (pi-PI3K), AKT (pi-AKT) and mTOR (pi-mTOR) decreased significantly, and the overexpression of PI3K and AKT reversed this change (Figure 3C–3F). Based on these results, we concluded that ITGB2 could activate the PI3K-AKT-mTOR pathway by mediating protein phosphorylation.

### ITGB2 promotes mitochondrial glycolytic transformation in ovarian cancer cells through the PI3K-AKT-mTOR axis

**Figure 4:**
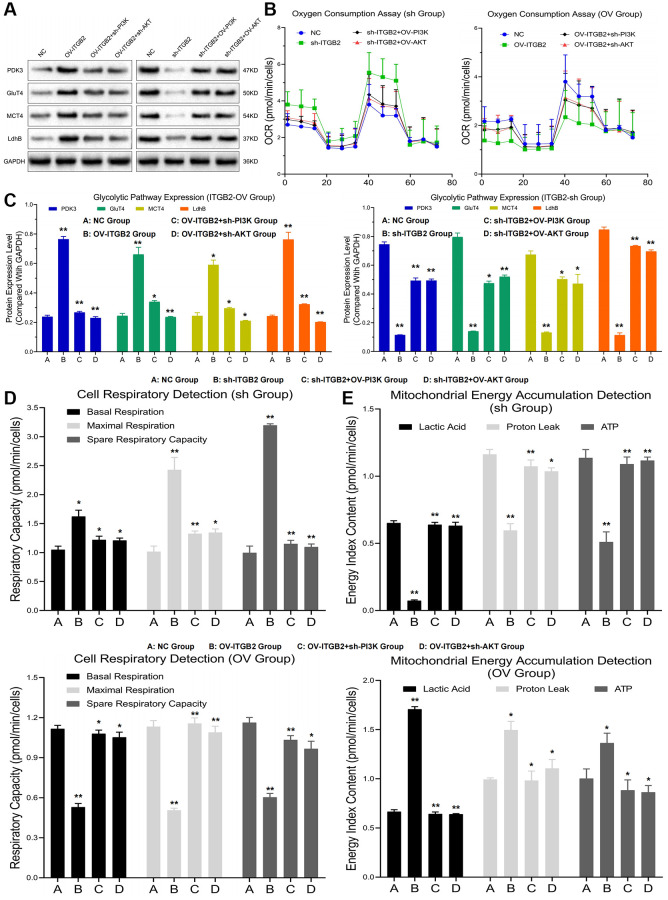
**Mitochondrial glycolysis indicators detection.** (**A**, **B**) Detection of glycolysis signal pathway expression and oxygen consumption in ovarian cancer cell models. (**C**) Data statistics of glycolysis signal pathway expression. (**D**) Mitochondrial respiration detection in ovarian cancer cell models. (**E**) Detection of mitochondrial energy metabolism in ovarian cancer cell models.

To explore the effect of ITGB2-mediated PI3K-AKT-mTOR pathway regulation on mitochondrial glycolysis in ovarian cancer cells, we constructed ovarian cancer cell models with different ITGB2, PI3K and AKT expression levels. The Seahorse detection revealed that the levels of oxygen consumption, basal respiration, maximal respiration and spare respiratory capacity in the mitochondria of ITGB2 knockdown ovarian cancer cells increased significantly, while the levels of these indicators decreased after the overexpression of PI3K and AKT. Moreover, the levels of oxygen consumption, basal respiration, maximal respiration and spare respiratory capacity in the mitochondria of ITGB2 overexpressing ovarian cancer cells clearly decreased, while the levels of these indicators correspondingly increased after the inhibition of PI3K and AKT (Figure 4B–4D).

The mitochondrial energy metabolism detection showed that the levels of lactic acid, proton leakage and ATP in the mitochondria of ITGB2-overexpressing ovarian cancer cells increased significantly, while the levels of these indicators decreased after the silencing of PI3K and AKT. Moreover, the levels of lactic acid, proton leakage and ATP in the mitochondria of ITGB2 knockdown ovarian cancer cells clearly decreased, while the levels of these indicators correspondingly increased after the overexpression of PI3K and AKT (Figure 4E). Based on these results we concluded that ITGB2 could promote mitochondrial glycolysis and energy conversion in ovarian cancer cells through PI3K-AKT-mTOR axis.

To further demonstrate the regulatory mechanism of ITGB2 on mitochondrial glycolysis in ovarian cancer cells, we constructed ovarian cancer cell models with different signaling pathway expression levels. The results revealed that the expression of critical glycolytic enzymes (such as Glu, T4MCT4 and LdhB) in ITGB2 overexpressing ovarian cancer cells was obviously upregulated compared with that in the NC group, while the knockdown of PI3K and AKT reversed this change. At the same time, in ITGB2 knockdown ovarian cancer cells the expression of these enzymes obviously decreased, while the overexpression of PI3K-AKT could rescue this process (Figure 4A, 4C). Thus, we concluded that ITGB2 signal pathway (PI3K-AKT-mTOR) could enhance mitochondrial glycolysis in ovarian cancer cells through upregulating rate-limiting enzymes.

### ITGB2 maintains mitochondrial stability and inhibits autophagy in ovarian cancer cells via the PI3K-AKT-mTOR axis

**Figure 5:**
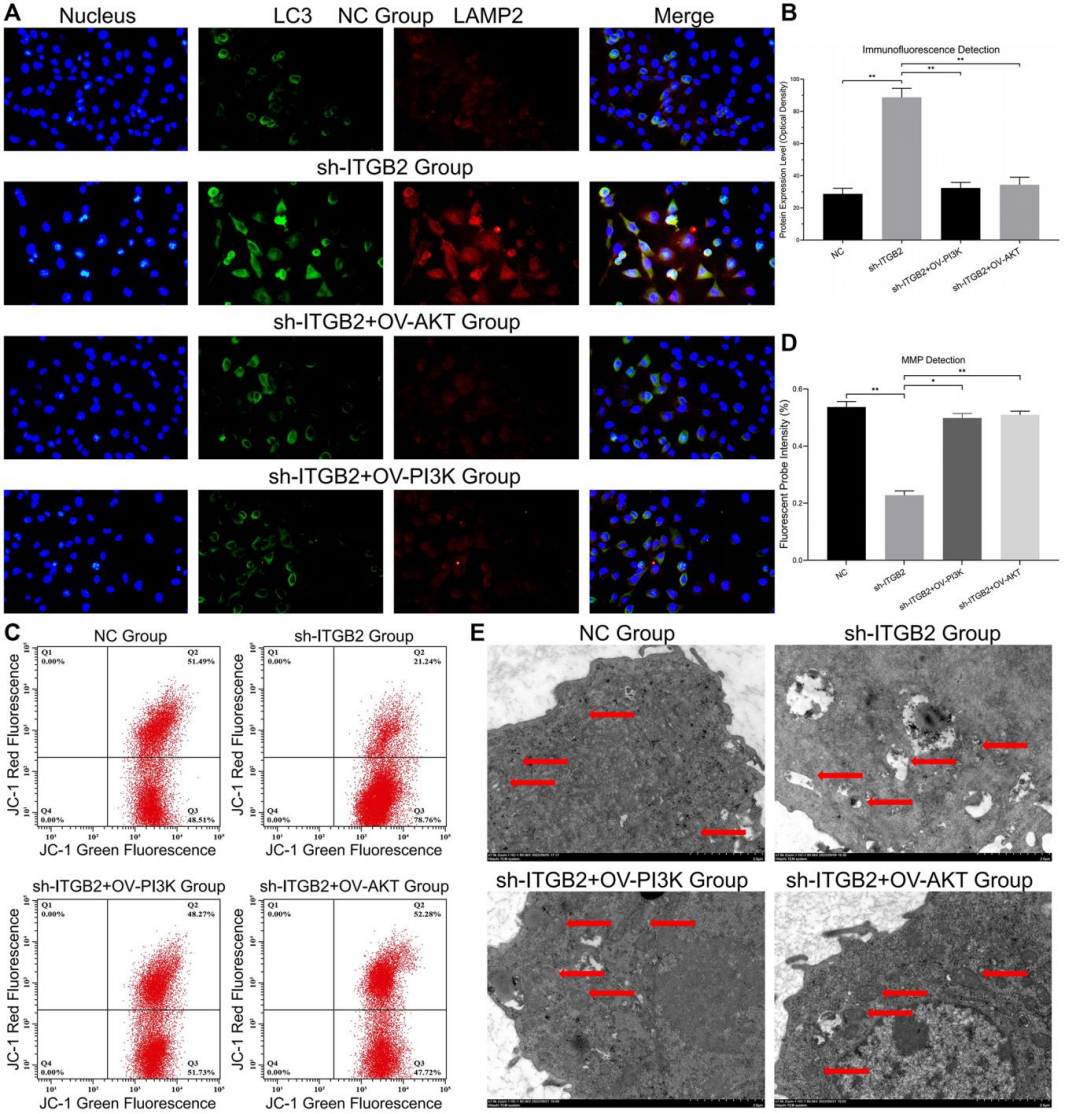
**Mitophagy detection in ovarian cancer cells.** (**A**, **B**) Immune co-localization detection of mitochondrial autophagy markers in ovarian cancer cell models and data statistics. (**C**, **D**) MMP detection in ovarian cancer cell models and data statistics. (**E**) TEM observation of mitophagy in ovarian cancer cells with different ITGB2 signal pathway expression.

Our findings revealed a simultaneous presence of mitophagy and lysosome markers, indicating a clear rise in the joint presence of LC3 and LAMP2 in ovarian cancer with reduced ITGB2 levels, while PI3K/AKT up-regulation could save this pathological change (Figure 5A, 5B). Then we used JC-1 probes to measure the MMP in ovarian cancer cells. The results showed that compared with the NC group, the ITGB2 knockdown group showed clearly decreased red fluorescence intensity, and these changes could be reversed by the overexpression of PI3K and AKT (Figure 5C, 5D). Concurrently, TEM analysis of mitochondrial structure revealed that in ovarian cancer cells with ITGB2 knockdown, there was a noticeable decrease in mitochondrial size, rupture of the mitochondrial membrane, and vanishing of mitochondrial cristae, in contrast to normal cells (Figure 5E). Consequently, it was deduced that ITGB2 has the potential to suppress mitophagy and preserve the steadiness of MMP in ovarian cancer cells via PI3K/AKT/mTOR signaling route.

### ITGB2 promotes the proliferation and invasion of ovarian cancer cells via the PI3K-AKT-mTOR axis

**Figure 6:**
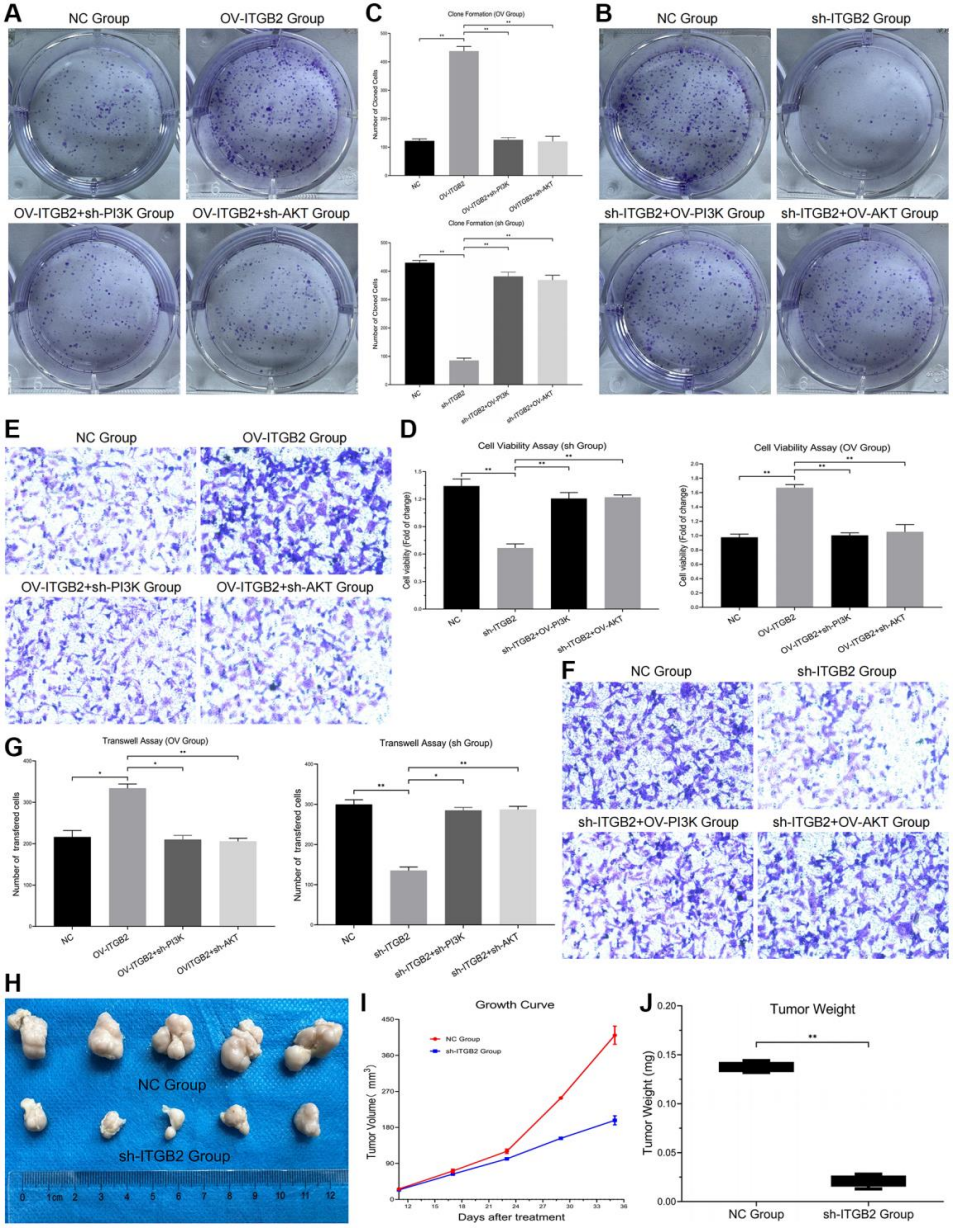
**Detection of malignant phenotypes in ovarian cancer cells.** (**A**–**C**) Clone Formation Assay in ovarian cancer cell models and data statistics. (**D**) CCK8 Assay in ovarian cancer cell models and data statistics. (**E**–**G**) Transwell Assay in ovarian cancer cell models and data statistics. (**H**) Tumor formation experiment *in vivo*. (**I**) Tumor growth measurement and data statistics. (**J**) Tumor weight detection and data statistics.

Experiments on colony formation revealed a noticeable rise in the count of newly formed ovarian cancer cells in the ITGB2 overexpressing group, contrasting with a corresponding decline following the inhibition of PI3K and AKT. Moreover, in the ITGB2 knockdown group, the number of newly generated ovarian cancer cells obviously decreased, and this number increased after PI3K/AKT overexpression (Figure 6A–6C). Our results suggested that ITGB2 might promote cell proliferation via the PI3K-AKT-mTOR signaling pathway in ovarian cancer cells.

We developed ovarian cancer cell models with different signal pathway expression through plasmid transfection, and detected tumor cell activity by CCK-8 Test. The results showed that cell activity was obviously inhibited in ITGB2 knockdown group, while this change could be reversed by PI3K/mTOR overexpression. Moreover, in ITGB2 overexpressing group the cell activity was obviously upregulated, and this change could be rescued through PI3K/mTOR knockdown. These results confirmed that ITGB2 could promote cell activity through the PI3K-AKT-mTOR signaling pathway in ovarian cancer cells (Figure 6D).

To clarify the effect of ITGB2 on the invasion of ovarian cancer cells and its possible mechanism, we used a Transwell test to evaluate changes in their invasive ability. The results showed that the number of ovarian cancer cells that crossed the basement membrane increased significantly in the ITGB2 overexpressing group, while it decreased after PI3K/AKT inhibition. Moreover, the number of invasive cells in the ITGB2 knockdown group obviously decreased, and it correspondingly increased after the overexpression of PI3K/AKT (Figure 6E–6G). Therefore, we concluded that ITGB2 could promote the invasion ability of ovarian cancer cells via the PI3K/AKT/mTOR pathway.

### Knockdown of ITGB2 inhibits the tumorigenicity in ovarian cancer cells

Here, we developed a xenograft mouse model to determine whether ITGB2 affects the oncogenic progression of ovarian cancer cells (SKOV3) *in vivo*. Normal SKOV3 cells and SKOV3 cells with ITGB2 knockdown were used to construct mouse xenograft models through subcutaneous inoculation. Findings indicated a notable decrease in both tumor size and mass in the ITGB2 knockdown group, in contrast to the NC group (Figure 6H–6J). Based on these results, we concluded that ITGB2 could promote the tumor growth of ovarian cancer cells through the PI3K- AKT-mTOR pathway *in vivo*.

## DISCUSSION

Integrin is a transmembrane adhesion receptor that has α and β subunits. This receptor interacts with factors of the extracellular matrix (including cadherin, immunoglobulin and selectin). Integrin-β is a group of integrins that play important roles in cell adhesion, proliferation and differentiation. ITGB2 (CD18) is a key subunit of the β2 integrin in tumor cells that can participate in cell adhesion matrix remodeling and signal transduction between tumor cells and the microenvironment, thus inducing infiltration, angiogenesis and specific immune responses in tumor cells [15–17].

Previous studies have shown that ITGB2 is involved in the occurrence, invasion and metastasis of various cancer types (such as liver, colon, breast cancer and leukemia). Puerkaiti observed that ITGB2 expression was upregulated in TNBC, and its expression level was closely related to tumor stage, local metastasis and clinical prognosis. Another study revealed that ITGB2 expression was closely related to CLL and myeloma in hematological malignancies. Hutterer found that ITGB2 expression in CLL patients could be regulated by DNA methylation, thus promoting tumor cell growth. Researchers also found a significant correlation between ITGB2 and drug sensitivity in patients with AML, and patients in the high-risk group were resistant to immunotherapy and conventional chemotherapy [18–20]. The results of a bioinformatics analysis showed that ITGB2 expression levels were significantly correlated with overall survival time in ovarian cancer cases; therefore, several scholars suggest that ITGB2 might be involved in the progression of ovarian cancer. Our research showed that ITGB2 expression was upregulated and that its downstream signaling pathway (PI3K-AKT-mTOR) was activated in ovarian cancer samples. This regulatory network was closely related to the malignancy of ovarian cancer samples. Therefore, we concluded that the ITGB2-related axis plays an important role in regulating malignant phenotypes in ovarian cancer cells.

In eukaryotic cells, aerobic respiration is the main process for generating energy, but under exceptional circumstances, cells can obtain energy through glycolysis, which is an anaerobic fermentation process. During this process, glucose is converted into pyruvate, which leads to its gradual accumulation in the cytoplasm and conversion to lactic acid, finally reducing the level of citric acid in mitochondria. Previous studies showed that excess accumulation of citrate in mitochondria could inhibit cell proliferation. Otto Warburg first found that cancer cells could increase glucose consumption and produce lactic acid under aerobic conditions. This reprogrammed cell metabolism is called the Warburg effect, which is considered an important marker in malignant tumors [4, 5]. Several scholars believe that the Warburg effect may act as a regulatory mechanism to avoid excessive accumulation of citrate in the mitochondria of malignant tumor cells and ultimately promote their proliferation and invasion and inhibit apoptosis. In this research we demonstrated that ITGB2 could promote glycolytic transformation through activating PI3K-AKT-mTOR axis in ovarian cancer cells.

Moreover, several scholars have found that nicotinamide adenine dinucleotide and oxidative phosphorylation are very important for the occurrence and development of malignant tumors. Santidrian showed that a decrease in NAD+/NADH expression enhanced tumor cell invasion and metastasis. Moreover, it was found that increasing the ratio of NAD+/NADH with certain compounds induced cell death. However, the regulatory mechanism of NAD+/NADH is still unclear [21]. Zhang found that ITGB2 promoted glycolysis and lactic acid secretion through the PI3K-AKT-mTOR pathway in cancer-associated fibroblast cells and enhanced NADH-dependent OXPHOS function in mitochondria through lactic acid oxidation. Thus, ATP production is increased and proliferation is promoted in oral squamous cell carcinoma. Several studies have suggested that the metabolism of ovarian cancer cells is heterogenous, including both aerobic glycolysis and oxidative phosphorylation processes [22–26]. These scholars believed that this process may be closely related to the progression of ovarian cancer. However, the effect of mitochondrial aerobic glycolysis (the Warburg effect) on malignant phenotypes in ovarian cancer and its possible regulatory mechanism have not been reported. Our results showed that ITGB2 related signal pathway (PI3K-AKT-mTOR) could promote cellular energy supply through mediating glycolytic transformation, thus inhibited mitophagy and maintained mitochondrial stability, finally encouraged the growth and infiltration of ovarian cancer cells. Moreover, this regulatory network could promote the growth of ovarian cancer cells *in vivo*.

## CONCLUSION

In this research we successfully proved that ITGB2 expression could promote glycolytic transformation in mitochondria through activating PI3K-AKT-mTOR signal pathway, thereby preserving mitochondrial stability and fostering malignant characteristics in ovarian cancer cells. Our study revealed the regulation of ITGB2-mediated glycolysis in mitochondrial respiratory chain operation and cellular energy supply, investigated how it impacts the cancerous characteristics of ovarian cancer cells, and finally provided an experimental basis for exploring new therapeutic targets for this disease.
